# CB2 Receptor Activation Ameliorates the Proinflammatory Activity in Acute Lung Injury Induced by Paraquat

**DOI:** 10.1155/2014/971750

**Published:** 2014-05-22

**Authors:** Zhenning Liu, Yu Wang, Hongyu Zhao, Qiang Zheng, Li Xiao, Min Zhao

**Affiliations:** Department of Emergency Medicine, Shengjing Hospital of China Medical University, No. 36 Sanhao Street, Heping District, Shenyang, Liaoning 110004, China

## Abstract

Paraquat, a widely used herbicide, is well known to exhibit oxidative stress and lung injury. In the present study, we investigated the possible underlying mechanisms of cannabinoid receptor-2 (CB2) activation to ameliorate the proinflammatory activity induced by PQ in rats. JWH133, a CB2 agonist, was administered by intraperitoneal injection 1 h prior to PQ exposure. After PQ exposure for 4, 8, 24, and 72 h, the bronchoalveolar lavage fluid was collected to determine levels of TNF-**α** and IL-1**β**, and the arterial blood samples were collected for detection of PaO_2_ level. At 72 h after PQ exposure, lung tissues were collected to determine the lung wet-to-dry weight ratios, myeloperoxidase activity, lung histopathology, the protein expression level of CB2, MAPKs (ERK1/2, p38MAPK, and JNK1/2), and NF-**κ**Bp65. After rats were pretreated with JWH133, PQ-induced lung edema and lung histopathological changes were significantly attenuated. PQ-induced TNF-**α** and IL-1**β** secretion in BALF, increases of PaO_2_ in arterial blood, and MPO levels in the lung tissue were significantly reduced. JWH133 could efficiently activate CB2, while inhibiting MAPKs and NF-**κ**B activation. The results suggested that activating CB2 receptor exerted protective activity against PQ-induced ALI, and it potentially contributed to the suppression of the activation of MAPKs and NF-**κ**B pathways.

## 1. Introduction

Paraquat (PQ) poisoning is by far one of the most clinically significant herbicides in terms of morbidity and mortality. The main target organ for acute PQ toxicity is the lung as a consequence of its accumulation against a concentration gradient, through the highly developed polyamine uptake system, and due to its capacity to generate huge amounts of prooxidant reactive species through a strenuous redox-cycle pathway [1]. Besides, another cytotoxic effect of PQ is the outbreak of progressive inflammation evoked by reactive oxygen species (ROS). During the inflammatory response in lung, immune cells including macrophages, neutrophils, and lymphocytes become activated, releasing IL-1*β*, TNF-*α*, and so on. It is pathologically characterized by lung edema, hemorrhage, interstitial inflammation, and bronchial epithelial cell proliferation [2]. Respiratory failure as a result of lung injury is the most common cause of death from PQ. Since the oxidative damage and the consequent inflammatory response are the major contributors to the toxic effects of PQ, finding an effective and feasible option for preventing or treating the consequent damage becomes an urgent problem.

The endogenous cannabinoid system is composed of the cannabinoid receptor types 1 and 2 (CB1 and CB2), the endogenous ligands (endocannabinoids), and enzymes that synthesize and degrade endocannabinoids. CB2 is mainly expressed in immune cells including neutrophils, eosinophils, monocytes, and natural killer cells [3–6]. Activation of the cannabinoid-2 (CB2) receptors by endocannabinoids [7], or selective synthetic agonists, has been shown to protect against tissue damage in various experimental models of ischemic-reperfusion injury [8, 9], atherosclerosis/cardiovascular inflammation [10], and other disorders by limiting inflammatory cell chemotaxis/infiltration, activation, and interrelated oxidative/nitrosative stress [11–14]. However, there are no reports that activating CB2 receptor may exert protective effects on PQ-induced acute lung injury and its potential biological mechanisms at present. In the present study, we investigated whether activating CB2 receptor has protective effects on PQ-induced acute lung injury in a rat model. In particular, we sought to further explore the role of the CB2 receptor in the process and investigate its underlying mechanisms.

## 2. Materials and Methods

### 2.1. Animals

Male Sprague-Dawley rats weighing 200–250 g were purchased from the Experimental Animal Center of China Medical University. Rats were housed in cages in a temperature-controlled (20–25°C) and humidity-controlled (40–70%) environment with a daily light-dark cycle. The rats had access to food and water ad libitum. All animal experiments were conducted in accordance with the Institutional Animal Ethics Committee and Animal Care Guidelines of China Medical University governing the use of experimental animals.

### 2.2. Reagents

PQ was purchased from Sigma-Aldrich (St. Louis, MO, USA). CB2R-agonist JWH133 was obtained from Enzo Life Sciences Ltd. (UK). Antibodies to p38MAPK, phosphor-p38MAPK, JNK1/2, phosphor-JNK1/2, ERK1/2, phosphor-ERK1/2, NF-*κ*Bp65, I*κ*B-*α*, phosphor-I*κ*B*α*, and *β*-actin were purchased from Cell Signaling Technology Inc. (Beverly, MA, USA). Rabbit anti-CB2 receptor polyclonal antibody was purchased from Santa Cruz Biotechnology Inc. (Santa Cruz, CA). The rat MPO determination kit was purchased from Jiancheng Bioengineering Institute of Nanjing (Nanjing, China). The ELISA kits for TNF-*α* and IL-1*β* were purchased from R&D system (R&D, USA).

### 2.3. Experimental Protocols

After adapting to the environment, the rats were randomly distributed into four groups: control group (1 mL normal saline solution, i.p. *n* = 24), PQ group (*n* = 24), low dose JWH133 (5 mg/kg, i.p.) + PQ group (*n* = 24), and high dose JWH133 (20 mg/kg, i.p.) + PQ group (*n* = 24). JWH133 was intraperitoneally administered at doses of 5 and 20 mg/kg. After 1 h, PQ was intraperitoneally administered at a dose of 20 mg/kg according to our previous research [15]. At 4, 8, 24, and 72 h after PQ administration, 6 rats were taken from each group at each endpoint for testing. Throughout the study period, each rat was observed carefully for clinical signs of PQ-related toxicity.

### 2.4. PaO_2_ Analysis in Arterial Blood

Arterial blood samples collected from the carotid arteries of the rats at 4, 8, 24, and 72 h after PQ administration were analyzed using a standard blood gas analyzer (ABL 505; Radiometer, Copenhagen, Denmark).

### 2.5. Wet-to-Dry (W/D) Lung Weight Ratios

The rats were killed via exsanguination 72 h after PQ administration. The whole lungs were removed. Each lung was dried, weighed, and then placed in an oven at 80°C for 48 h to obtain the “dry” weight. The ratio of the weight of the wet lung to the weight of the dry lung was calculated to assess tissue edema [16].

### 2.6. Histopathologic Evaluations

The rats were killed 72 h after PQ administration. The lungs were then removed and stored in 4% paraformaldehyde for 48 h at 4°C. Hematoxylin and eosin staining was carried out according to the regular staining method, and the slides were evaluated using a semiquantitative scoring method. Lung injury was graded in a blinded fashion from 0 (normal) to 4 (severe) for interstitial inflammation, neutrophil infiltration, congestion, and edema. The total lung injury score was calculated by adding up the individual scores from each category [16].

### 2.7. BALF Collection

At 4, 8, 24, and 72 h after PQ administration, rats were euthanized and BALF was performed on the left lung with 4 mL phosphate-balanced saline solution in 2.5 mL aliquots after cannulation of the left trachea. The collected BALF was centrifuged at 1000 g for 10 min; the supernatant was collected and stored at −80°C, for later TNF-*α* and IL-1*β* levels measurement.

### 2.8. TNF-*α* and IL-1*β* ELISA Assays

The levels of TNF-*α* and IL-1*β* in the BALF were measured via ELISA assays using commercially available kits according to the manufacturer's recommended instructions. The levels of TNF-*α* and IL-1*β* in the samples were calculated based on a standard curve. The detection ranges of the TNF-*α* and IL-1*β* ELISA assays were 12.5–800 pg/mL and 31.25–2000 pg/mL, respectively. Samples that had a concentration that exceeded the limit of the standard curve were measured after dilution.

### 2.9. Pulmonary MPO Activity

The activity of MPO was determined in the lung tissue by MPO kit (Nanjing Jiancheng, China). At 4, 8, 24, and 72 h after PQ treatment, rats were killed and the left lungs were excised. One hundred milligrams of lung tissue was homogenized and fluidized in extraction buffer to obtain 5% of the homogenate. The sample including 0.9 mL homogenate and 0.1 mL of reaction buffer was heated to 37°C in a water bath for 15 min, and then the enzymatic activity was determined by measuring the changes in absorbance at 460 nm using a 96-well plate reader and expressed as U/g weight.

### 2.10. Immunohistochemistry

The lung tissues were fixed in 4% paraformaldehyde for 48 h at 4°C and processed for paraffin embedding. Paraffin-embedded blocks were cut into 4 *μ*m thick sections and mounted onto slides. The sections were pretreated at 60°C for 1 h, then dewaxed in xylene, hydrated, and washed in 0.01 mol/L of citrate buffer. After inhibiting endogenous peroxidase using 3% H_2_O_2_ in methanol, the sections were incubated with anti-CB2R polyclonal antibody overnight at 4°C. The sections were then thoroughly washed with a phosphate-buffered saline (PBS) solution, after which point the corresponding secondary antibodies were applied and incubated at room temperature for 30 min. Reaction products were visualized following incubation with diaminobenzidine (DAB) and then counterstained with hematoxylin. Negative controls were generated by omitting the primary antibodies.

### 2.11. Western Blot Analysis

Lung tissue samples were harvested and frozen in liquid nitrogen immediately until homogenization. Proteins were extracted from the lungs using a Nuclear and Cytoplasmic Protein Extraction Kit (Beyotime Biotechnology, China) according to the manufacturer's protocol. To extract the total protein from lung tissue, protein concentrations were determined by BCA protein assay kit and equal amounts of protein were loaded per well on a 10% sodium dodecyl sulphate polyacrylamide gel (SDS-PAGE). Subsequently, proteins were transferred onto polyvinylidene difluoride membrane. The membranes were washed in Tris-buffered saline with Tween 20 and incubated in 5% skim milk (Sigma) at room temperature for 2 h on a rotary shaker and followed by TBS-T washing. Incubations with rabbit polyclonal antibodies specific for CB2 receptor, p38MAPK, phosphor-p38MAPK, JNK1/2, phosphor-JNK1/2, ERK1/2, phosphor-ERK1/2, NF-*κ*Bp65, I*κ*B-*α*, and phosphor-I*κ*B*α* in diluent buffer (5% skim milk in TBST) were performed overnight at 4°C. Then the membrane was washed with TBS-T followed by incubation with the peroxidase-conjugated secondary antibody at room temperature for 1 h. Immunoreactive bands were visualized with an enhanced chemiluminescence western blot kit in accordance with the manufacturer's instructions. The *β*-actin western blot was performed as the internal control of protein loading. The signals were detected with an enhanced chemiluminescence kit (Pierce) and exposed on X-ray film. After the film was scanned with a GS-700 imaging densitometer (Bio-Rad, Hercules, CA), a quantitative analysis was performed using Multi-Analyst software (Bio-Rad).

### 2.12. Statistical Analyses

The data are expressed as the means ± SD. Statistical analyses were carried out using SPSS 16.0. One-way ANOVA followed by the Student-Newman-Keuls test was used to compare the results that were obtained in the different treatment groups. Differences were considered to be statistically significant when *P* < 0.05.

## 3. Results

### 3.1. CB2 Activation Attenuates Lung W/D Ratios in Rats with PQ-Induced Acute Lung Injury

**Figure 1:**
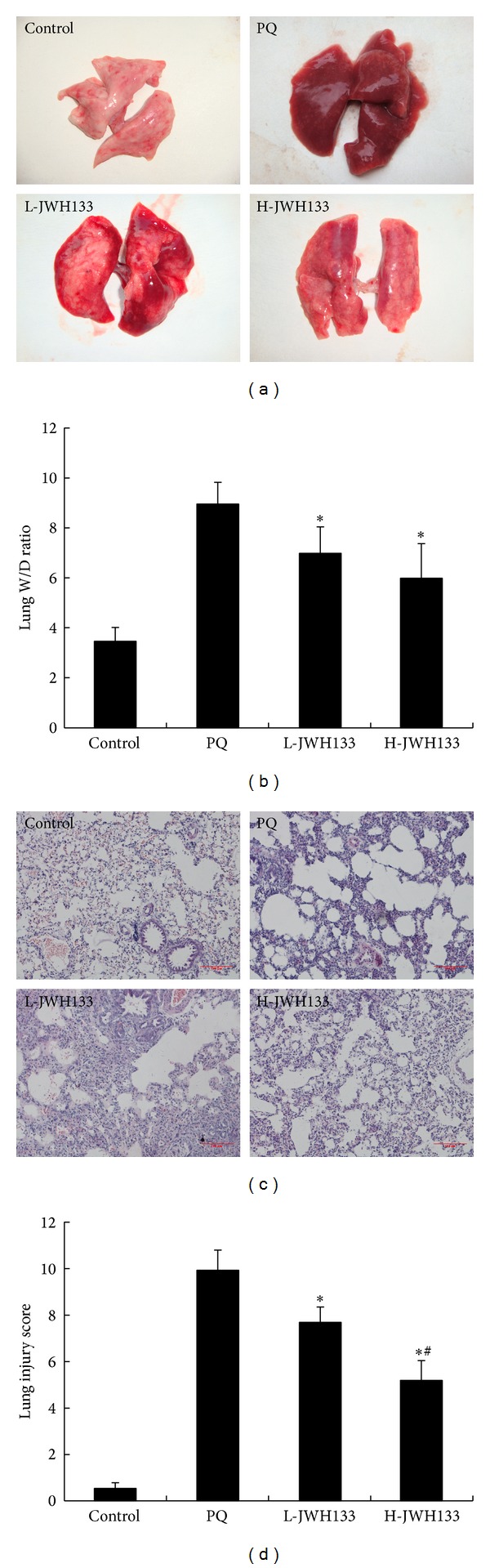
Effects of JWH133 on lung W/D ratio and histopathological changes in lung tissues of PQ-induced acute lung injury rats. JWH133 was intraperitoneally administered 1 h before intraperitoneal administration of PQ. The lung W/D ratio (a, b) and lung histological evaluation (c, d, HE staining) were determined after PQ administration for 72 h. L-JWH133: JWH133 at the low dose of 5 mg/kg and H-JWH133: JWH133 at the high dose of 20 mg/kg. The values presented are the mean ± SD. *P < 0.05 versus PQ group; ^#^ *P* < 0.05 versus L-JWH133 group.

The lung W/D ratio was determined in order to assess the severity of pulmonary edema. As shown in Figures 1(a) and 1(b), the lung W/D ratios in the PQ-treated group were significantly higher than those in the control group at 72 h after PQ administration (*P* < 0.05). CB2 activation decreased the lung W/D ratios in rats with PQ-induced lung injury (*P* < 0.05).

### 3.2. CB2 Activation Improves PQ-Induced Histological Damage

As shown in Figures 1(c) and 1(d), the lungs of rats which were exposed to PQ for 72 h displayed significant inflammatory alterations that were characterized by lung edema, alveolar hemorrhage, inflammatory cell infiltration, and destruction of alveolar structure. These effects were dramatically reduced and became more focal in JWH133-treated rats, especially for high dose. The lung injury scores were significantly decreased dose-dependently.

### 3.3. CB2 Activation Attenuates Markers of Acute Lung Injury (PaO_2_)

**Figure 2:**
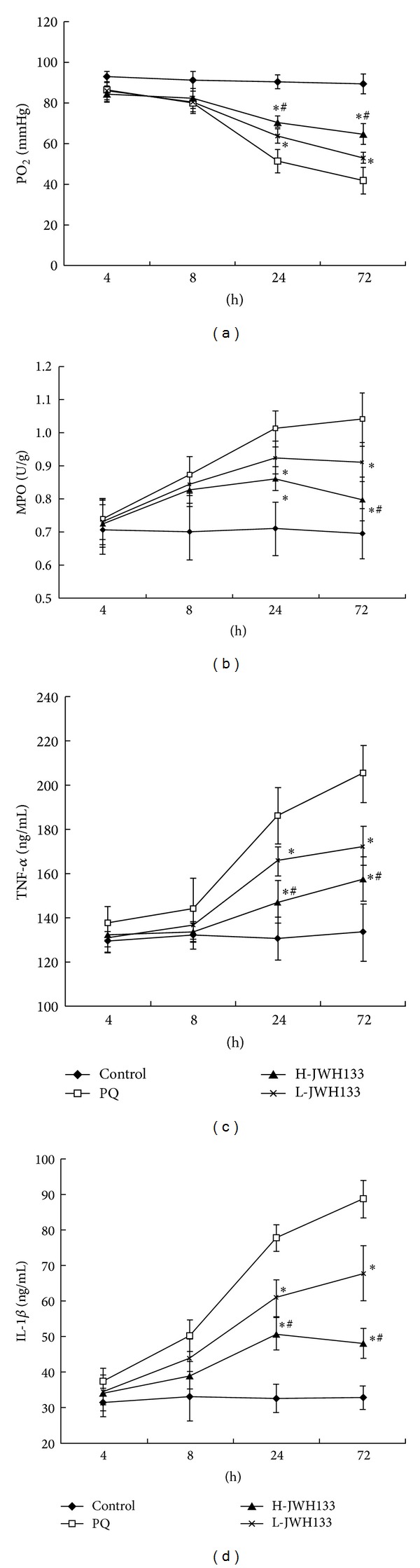
Effects of JWH133 on the PaO_2_ in the arterial blood, MPO activity, and cytokine (IL-1*β* and TNF-*α*) levels in the BALF of PQ-induced acute lung injury rats. JWH133 was intraperitoneally administered 1 h before intraperitoneal administration of PQ. The arterial blood and BALF were collected after PQ administration for 4, 8, 24, and 72 h to analyze PaO_2_ level (a), MPO activity (b), and levels of TNF-*α* and IL-1*β* (c, d). L-JWH133: JWH133 at the low dose of 5 mg/kg and H-JWH133: JWH133 at the high dose of 20 mg/kg. The values presented are the mean ± SD. *P < 0.05 versus PQ group; ^#^ *P* < 0.05 versus L-JWH133 group.

For assessments of acute lung injury induced by PQ, PaO_2_, derived from the arterial blood samples, was measured individually. As shown in Figure 2(a), after 4 h of PQ poisoning, PaO_2_ decreased gradually compared to the control rats. PaO_2_ declined obviously for the 24 h and went down to the valley value. Compared to the PQ group, the decline extent of PaO_2_ was palliative in the JWH133-treated group.

### 3.4. CB2 Activation Attenuates PQ-Induced Marked Neutrophil Infiltration

Neutrophils were important mediators of the delayed tissue injury after PQ administration. An indicator of neutrophil infiltration was the MPO activity. In PQ poisoning group, the lung MPO activity was obviously elevated time-dependently compared to the control group. In contrast, the marked increase in lung MPO activity induced by PQ was largely attenuated by JWH133, especially for high dose JWH133 (Figure 2(b)).

### 3.5. CB2 Activation Attenuates PQ-Induced Proinflammatory Cytokine Secretion

The effects of JWH133 on the production of TNF-*α* and IL-1*β* in the BALF at 4, 8, 24, and 72 h after PQ administration were analyzed with ELISA. As shown in Figures 2(c) and 2(d), treatment with PQ alone caused significant increases in TNF-*α* and IL-1*β* compared to the control group (*P* < 0.05). JWH133 markedly reduced the production of TNF-*α* and IL-1*β* at 24 or 72 h after PQ administration compared to the PQ-treated group (*P* < 0.05). Compared to the L-JWH133 group, the effect was more significant in the H-JWH133 group.

### 3.6. CB2 Receptor Expression in the Lung Tissues of Rats with PQ-Induced Acute Lung Injury

**Figure 3:**
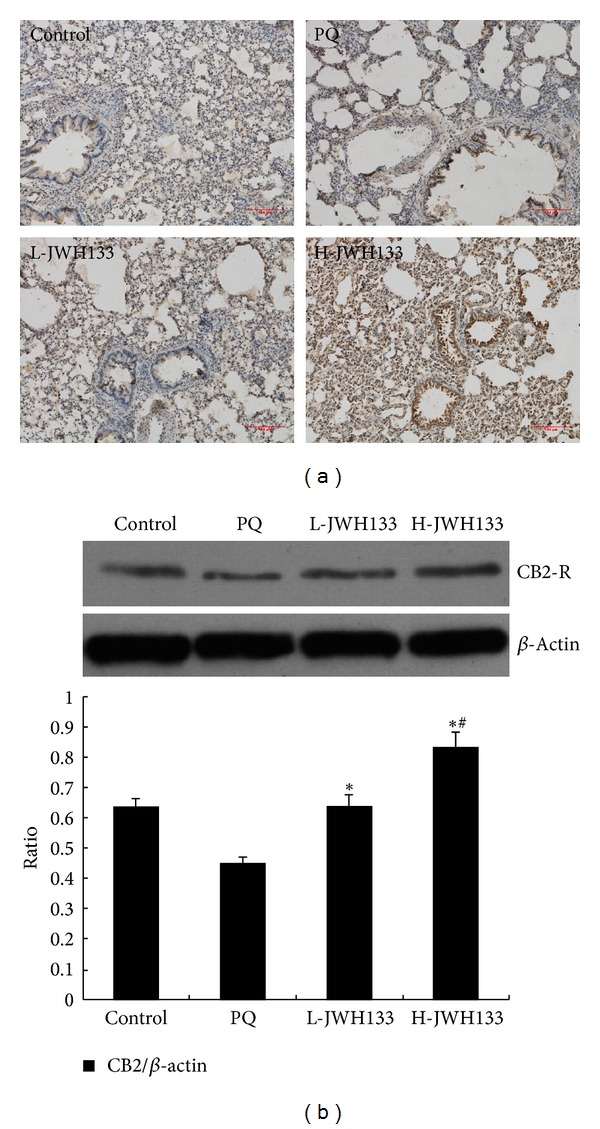
Effects of JWH133 on CB2 receptor in lung tissues of paraquat- (PQ-) induced acute lung injury rats. JWH133 was intraperitoneally administered 1 h before intraperitoneal administration of PQ. Lung tissue was collected after PQ administration for 72 h to determine the expression of CB2 receptor by immunohistochemistry (a) and Western blotting (b). L-JWH133: JWH133 at the low dose of 5 mg/kg and H-JWH133: JWH133 at the high dose of 20 mg/kg. The values presented are the mean ± SD. *P < 0.05 versus PQ group; ^#^ *P* < 0.05 versus L-JWH133 group.

CB2 receptor expression in the lung tissues was illustrated in Figure 3(a). In the control group, CB2 receptor positive signals were weakly observed in the lung tissues. At 72 h after PQ administration, CB2 receptor positive signals were further decreased in the lung tissues. Notably, JWH133 pretreatment significantly increased CB2 receptor positive signals in the lung tissues. The result of the western blotting analysis was illustrated in Figure 3(b). The PQ-treated group revealed decreased CB2 receptor expression as compared to the control group. JWH133 pretreatment significantly increased CB2 receptor expression in the lung tissues in a dose-dependent manner compared to the PQ-treated group (*P* < 0.05).

### 3.7. CB2 Activation Attenuates PQ-Induced p38MAPK, ERK1/2, and JNK1/2 Activation in Lung Tissue of Rats with PQ-Induced Acute Lung Injury

**Figure 4:**
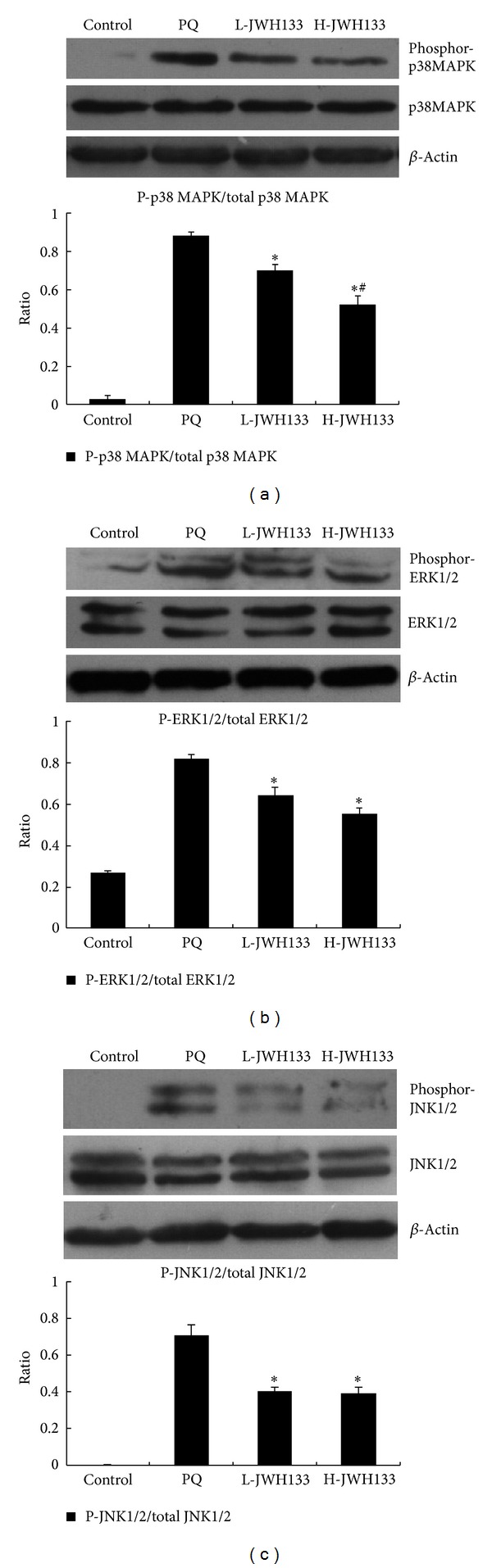
Effects of JWH133 on p38 MAPK, ERK1/2, and JNK1/2 signal transduction in lung tissues of PQ-induced acute lung injury rats. JWH133 was intraperitoneally administered 1 h before intraperitoneal administration of PQ. Lung tissue was collected after PQ administration for 72 h to determine the expression of p38 MAPK, ERK1/2, and JNK1/2 by Western blotting. L-JWH133: JWH133 at the low dose of 5 mg/kg and H-JWH133: JWH133 at the high dose of 20 mg/kg. The values presented are the mean ± SD. *P < 0.05 versus PQ group; ^#^ *P* < 0.05 versus L-JWH133 group.

As shown in Figure 4, there was a marked increase in PQ-induced phosphor-p38MAPK, phosphor-ERK1/2, and phosphor-JNK1/2 in the lung tissues. Pretreatment with JWH133 attenuated p38MAPK, ERK1/2, and JNK1/2 increases and inhibited the MAPKs phosphorylation compared to the PQ-treated group (*P* < 0.05). However, only p38MAPK phosphorylation was inhibited by JWH133 in a dose-dependent manner.

### 3.8. CB2 Activation Attenuates NF-*κ*B Signal Transduction in the Lung Tissues of Rats with PQ-Induced Acute Lung Injury

**Figure 5:**
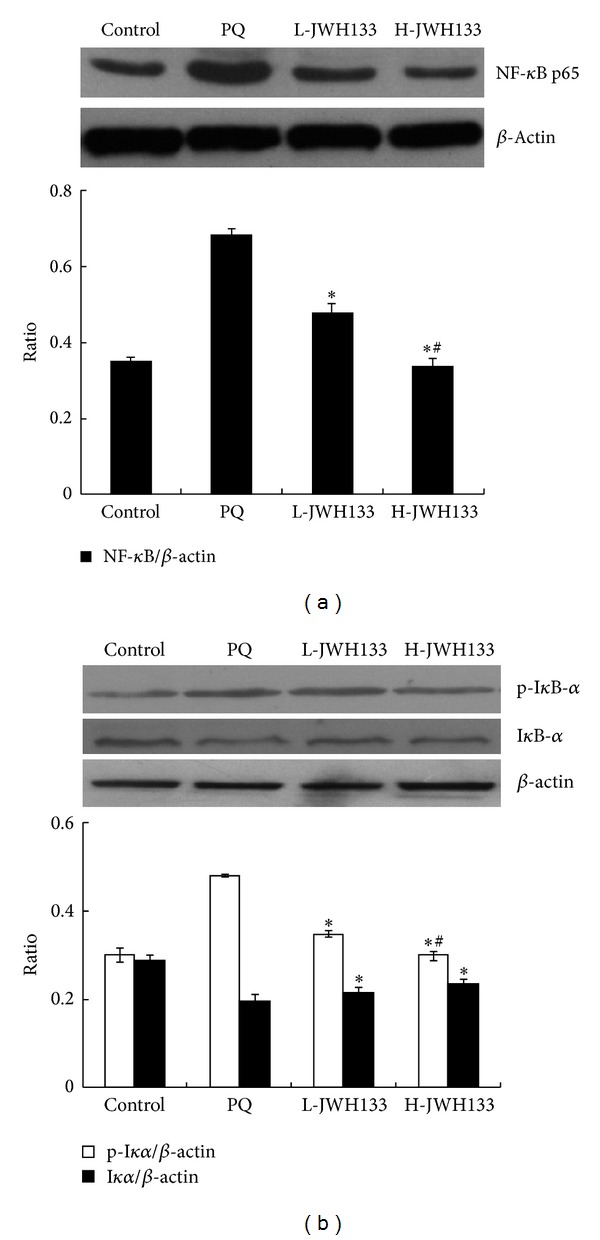
Effects of JWH133 on NF-*κ*B signal transduction in lung tissues of PQ-induced acute lung injury rats. JWH133 was intraperitoneally administered 1 h before intraperitoneal administration of PQ. Lung tissue was collected after PQ administration for 72 h to determine the expression of NF-*κ*B p65, I*κ*B-*α*, and pI*κ*B-*α* by Western blotting. L-JWH133: JWH133 at the low dose of 5 mg/kg and H-JWH133: JWH133 at the high dose of 20 mg/kg. The values presented are the mean ± SD. *P < 0.05 versus PQ group; ^#^ *P* < 0.05 versus L-JWH133 group.

As shown in Figure 5(a), the PQ-treated group displayed significant increase in the levels of the p65 subunit of NF-*κ*B in the nuclear extracts. In contrast, JWH133 treatment significantly decreased the levels of the p65 subunit of NF-*κ*B dose-dependently (*P* < 0.05). As shown in Figure 5(b), the PQ-treated group displayed significant I*κ*B-*α* degradation and phosphorylation compared to the control group. In contrast, I*κ*B-*α* degradation and phosphorylation in the JWH133-pretreated groups were significantly reduced compared to the PQ-treated group (*P* < 0.05).

## 4. Discussion

Acute respiratory distress syndrome (ARDS) and acute lung injury (ALI) are the major causes of mortality and morbidity in PQ poisoning patients. Histologically, the acute exudative phase (the first 24–72 h) of ALI is characterized by infiltration of inflammatory cells and disruption of the alveolar-capillary barrier, leading to a proteinaceous exudate that floods the alveolar spaces and then impairs gas exchange and precipitates respiratory failure [17, 18]. Arterial partial pressure of oxygen (PaO_2_) and lung wet-to-dry weight (W/D) ratio can be used as indicators of lung injury according to previous reports [19, 20]. To investigate the effects of JWH133 on PQ-induced acute lung injury, we detected the lung W/D ratio of sampled lung tissues and PaO_2_ in arterial blood samples. The lung W/D ratio in the JWH133-pretreated group was significantly lower than that in the PQ-treated group. The PaO_2_ in arterial blood in the JWH133-pretreated group was higher than that in the PQ-treated group. Our experiments results suggested that treatment with JWH133 significantly attenuated lung edema and improved lung function. We also observed PQ-induced pathological alterations, including alveolar edema, hemorrhage, inflammatory cell infiltration, and diffuse alveolar collapse that was accompanied by wall thickening. However, pretreatment with JWH133 was very effective at preventing PQ-induced lung tissue damage in rats. In ALI, the predominant inflammatory cells are the neutrophils, which play an important role in the development of most cases of ALI [21]. MPO is an enzyme located mainly in the primary granules of neutrophils and its main function is to kill microorganisms, but under certain conditions, it produces excess oxidant leading to tissue damage [22]. In our study, we found that the recruitment of neutrophils in the airways and MPO activity in the lungs were dramatically increased after PQ administration. In contrast, pretreatment of JWH133 significantly decreased MPO activity and reduced neutrophil infiltration.

Lung neutrophil recruitment and the subsequent lung injury are dependent upon the expression of the proinflammatory cytokines, such as TNF-*α* and IL-1*β* [23]. Both TNF-*α* and IL-1*β* are the early cytokines that are released from alveolar macrophages in the early inflammatory phases of lung injury [24, 25]. These cytokines, as well as other proinflammatory compounds, initiate, amplify, and perpetuate the inflammatory response during acute lung injury. TNF-*α* may play a role in the initiation or progression of multiple organ failure during endotoxic shock, and it has also been shown to be a particularly important mediator of acute lung injury [26]. IL-1*β* plays a key role in the development of acute lung injury and can inhibit fluid transport across the distal lung epithelium [27] causing surfactant abnormalities [28] and increasing protein permeability across the alveolar-capillary barrier [29]. In this experiment, we found that the levels of TNF-*α* and IL-1*β* were significantly increased in BALF of rats after PQ exposure. However, when the rats were pretreated with JWH133, the levels of TNF-*α* and IL-1*β* in BALF of PQ-poisoned rats were significantly decreased. These findings indicate that CB2 activation effectively inhibited PQ-induced lung injury and inflammatory cells infiltration in vivo.

Pulmonary expression of these mediators, especially TNF-*α* and IL-1*β*, has been linked to activation (nuclear translocation) of the transcription factor NF-*κ*B [30]. NF-*κ*B is normally sequestered in the cytoplasm by a family of inhibitory proteins known as I*κ*Bs. A wide variety of stimuli, which have been extensively studied over the past two decades, can cause I*κ*B*α* phosphorylation, a process that is followed by its ubiquitination and subsequent degradation. The loss of I*κ*B*α* results in the release of the free NF-*κ*B subunit p65, which translocates from the cytoplasm to the nucleus, where it triggers the transcription of multiple proinflammatory genes, including cytokines (TNF-*α*, IL-1*β*, iNOS, COX2, etc.), chemokines, and adhesion molecules [31, 32]. Indeed, not only proinflammatory mediators (bacterial LPS, TNF-*α*, IL-1*β*, MMPs, COX2, and inducible nitric oxide synthase) [33, 34] but also ROS can activate intracellular transcription factors NF-*κ*B. In our previous study [15], we demonstrated that PQ generate a great amount of ROS and activate NF-*κ*B in rats. Meanwhile, it was also proved that NF-*κ*B played an important role in lung injury in PQ-poisoned rats. Our results demonstrated that PQ induced I*κ*B-*α* degradation and phosphorylation, which increased the levels of the p65 subunit of NF-*κ*B in the nuclear extracts of lung tissues. In contrast, JWH133 treatment inhibited the degradation and phosphorylation of I*κ*B-*α* and decreased the levels of the p65 subunit of NF-*κ*B in the nuclear extracts of the lung tissues. Therefore, the potent anti-inflammatory effects of JWH133 may be suggested to involve I*κ*B-*α* activation, which precipitates the translocation of the p65 subunit from the cytoplasm to the nucleus. However, the cellular targets and molecular mechanisms that lead to JWH133-mediated I*κ*B-*α* activation remain to be elucidated.

MAPKs, which are a group of signaling molecules mainly consisting of three well-characterized subfamilies, involving ERK1/2, JNK1/2, and p38MAPK [35] are involved in signal transduction of extracellular hormones, growth factors, cytokines, and bacterial antigens and also play key roles in inflammatory reactions [36]. It has been found that p38 MAPK activation leads to increased expression of various cytokine genes including IL-1*β* [37], TNF-*α*, IL-8, and IL-6 [38, 39]. JNK1/2 and ERK1/2 inhibition has also shown efficacy in inhibiting the production of proinflammatory mediators [40]. Our results showed that MAPKs activities including ERK1/2, p38 MAPK, and JNK1/2 were all activated in the process of acute lung injury induced by PQ. However, pretreatment with JWH133 inhibited phosphorylation of ERK1/2, p38 MAPK, and JNK1/2, which suggested that JWH133 exerted the inhibitory effect on cytokine production by inhibiting the MAPKs activity.

Sustained ROS generation activated MAPKs (e.g., p38MAPK, ERK1/2, and JNK1/2) [41, 42] and proinflammatory pathways (e.g., NF-*κ*B) [43, 44] in various cell types, in turn remodulating/regulating important inflammatory and cell survival and death processes in the previous studies such as placent [45] and hepatic I/R (ischaemia-reperfusion) [14]. The activation of MAPK/NF-*κ*B pathway plays a crucial role in many aspects of immune mediated inflammatory responses [46]. It has been found that MAPK pathway activation can lead to activation of the transcription factor NF-*κ*B, which is known to be relevant to cytokine gene expression [47]. In addition, a recent study showed that silencing of JNK and p38 MAPK decreased NF-*κ*B phosphorylation and increased inhibitor of NF-*κ*B (I*κ*B) *α* levels, which attenuated oxidative stress, inflammation, and apoptosis furtherly [48]. In our study, JWH133 inhibited MAPKs activity, which suggested that JWH133 may activate I*κ*B-*α* and attenuate the NF-*κ*B-induced inflammatory response via inhabitation of MAPKs activity. However, this hypothesis remains to be further elucidated.

Overwhelming evidence has established an important role for endocannabinoid-CB2 receptor signaling in a large number of the major pathologies including inflammatory, autoimmune, cardiovascular, gastrointestinal, liver, kidney, neurodegenerative, psychiatric, and many other diseases [49]. CB2 receptor activation in general mediates immunosuppressive effects, which limit inflammation and associated tissue injury in large number of pathological conditions [49]. CB2 receptor agonists dramatically attenuate iNOS induction and ROS generation in LPS-activated microglia. These effects are due to their reduction of phosphorylation of ERK1/2 and activation of NF-*κ*B [50]. CB2 agonist inhibited LPS-induced proinflammatory cytokine expression in peripheral blood and attenuates LPS-stimulated ERK1/2 and JNK phosphorylation in monocytes [51]. JWH133 reduced expression of active p38 MAPK, JNK, and proinflammatory cytokines (IL-1*β*, IL-6, and TNF-*α*) induced cognitive improvement in a genetic mice model of Alzheimer's disease [52]. In the present study, we found that CB2 receptors were expressed in lung epithelial cells, endothelial cells, and immune cells. The CB2 receptors expression in PQ-poisoned rats was lower than that in control rats. It may be correlated with CB2 receptor internalization. However, there may be some other reasons for this phenomenon. Therefore, the underlying mechanism needs to be further investigated in the next experiment. Our results suggest that JWH133 may exert its effect on PQ-induced lung injury and it can regulate inflammation responses including MAPKs signal pathway and NF-*κ*B pathway via CB2 receptor activation. This hypothesis requires further verification in vitro.

## 5. Conclusions

In summary, we obtained the results that PQ administration reduced the PaO_2_ in the arterial blood, recruited leukocytes with the enhanced expression of cytokines (TNF-*α* and IL-1*β*), activated the MAPKs signaling including p38MAPK, ERK1/2, JNK1/2, and the nuclear transcription factor NF-*κ*B, and finally evoked inflammatory response with manifestation of alveolar structure damage, congestion, and edema in the lung tissue. These effects were attenuated by CB2 agonist JWH133. Moreover, NF-*κ*B activation was significantly suppressed and the phosphorylation of p38MAPK, ERK1/2, and JNK1/2 was substantially downregulated after JWH133 pretreatment. Taken together, the results suggest that activating CB2 receptor can inhibit the proinflammatory cytokine expression and may exert a protective effect on PQ-induced ALI, and it potentially contributes to the suppression of the activation of MAPKs and NF-*κ*B pathways. The specific biological mechanisms of MAPKs and NF-*κ*B pathways triggered by CB2 activation need to be further investigated in the next experiments using CB2-selective antagonists or knockout animals. These current results may only have important implications for the alleviation of acute lung injury caused by PQ.

## Abbreviations

PQ: Paraquat
CB2: Cannabinoid receptor-2
BALF: Bronchoalveolar lavage fluid
TNF-*α*: Tumor necrosis factor-*α*
IL-1*β*: Interleukin-1*β*
W/D: Wet-to-dry weight
MPO: Myeloperoxidase
ERK1/2: Extracellular signal-regulated kinase 1/2
p38MAPK: p38 Mitogen-activated protein kinase
JNK: c-Jun N-terminal kinase
NF-*κ*B: Nuclear factor-*κ*B
I*κ*B-*α*: Inhibitor of *κ*B protein-*α*
ROS: Reactive oxygen species
ALI: Acute lung injury
ARDS: Acute respiratory distress syndrome
PaO_2_: Arterial oxygen partial pressure.
