# Correction to “CB2 Receptor Activation Ameliorates the Proinflammatory Activity in Acute Lung Injury Induced by Paraquat”

**DOI:** 10.1155/bmri/9816458

**Published:** 2026-10-17

Z. Liu, Y. Wang, H. Zhao, Q. Zheng, L. Xiao, M. Zhao, “CB2 Receptor Activation Ameliorates the Proinflammatory Activity in Acute Lung Injury Induced by Paraquat,” *BioMed Research International*, 2014, 971750, https://doi.org/10.1155/2014/971750

In the article, concerns were reported regarding the *β*‐actin bands in Figures 3b, 4b and 5a being identical. Further investigation by the publisher also identified that the third and fourth CB2‐R bands in Figure 3b appear highly similar to the third and fourth Phosphor‐p38MAPK bands in Figure 4a, after being flipped horizontally.

The authors confirmed that for the *β*‐actin bands, these were generated from the same blotting membrane, which was stripped and re‐probed with different antibodies. They have explained that the similarity between the CB2‐R bands in Figure 3b and the Phosphor‐p38MAPK bands in Figure 4a was introduced due to image misuse during figure preparation.

The authors repeated the experiments and provided new Western blots, which after assessment by the Chief Editor have been deemed acceptable for correction. Please find the corrected Figures 3, 4 and 5 below, which contain:

• New blots for the expression of Phosphor‐p38MAPK and Total‐p38MAPK using Western blotting,
• New immunoblotting analyses for CB2‐R, Phosphor‐ERK1/2, Total‐ERK1/2, and NF‐*κ*B p65 to address the initial *β*‐actin bands concern

**Figure 3:**
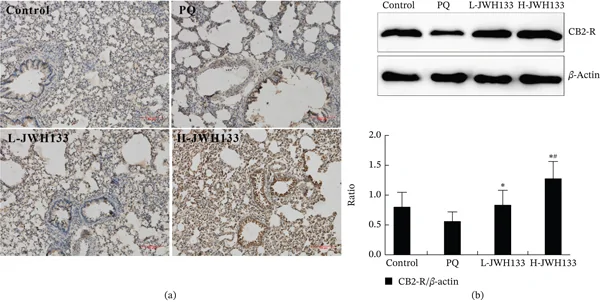
Effects of JWH133 on CB2 receptor in lung tissues of paraquat (PQ)‐induced acute lung injury rats. JWH133 was intraperitoneally administered 1 h before intraperitoneal administration of PQ. Lung tissue was collected after PQ administration for 72 h to determine the expression of CB2 receptor by (a) immunohistochemistry and (b) Western blotting. L‐JWH133: JWH133 at the low dose of 5 mg/kg and H‐JWH133: JWH133 at the high dose of 20 mg/kg. The values presented are the mean ± SD. ^*^ *p* < 0.05 versus PQ group; ^#^ *p* < 0.05 versus L‐JWH133 group.

**Figure 4:**
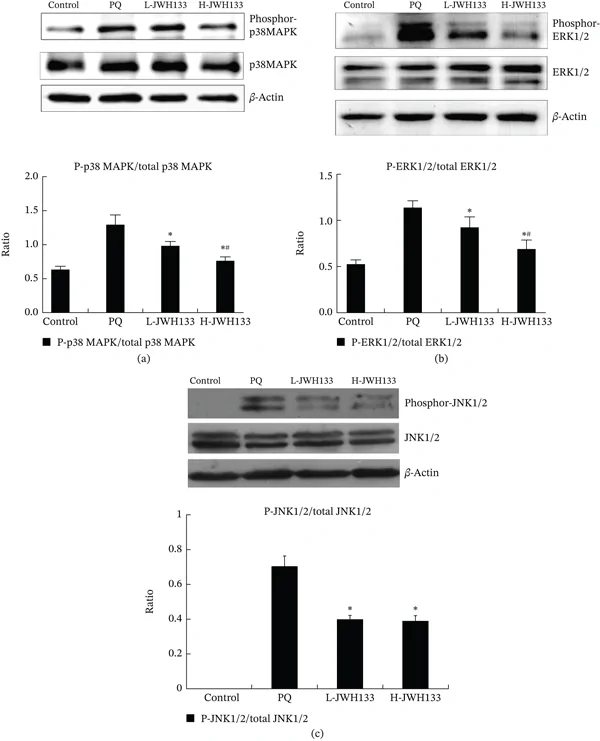
Effects of JWH133 on p38 (a) MAPK, (b) ERK1/2, and (c) JNK1/2 signal transduction in lung tissues of PQ‐induced acute lung injury rats. JWH133 was intraperitoneally administered 1 h before intraperitoneal administration of PQ. Lung tissue was collected after PQ administration for 72 h to determine the expression of p38 MAPK, ERK1/2, and JNK1/2 by Western blotting. L‐JWH133: JWH133 at the low dose of 5 mg/kg and H‐JWH133: JWH133 at the high dose of 20 mg/kg. The values presented are the mean ± SD. ^*^ *p* < 0.05 versus PQ group; ^#^ *p* < 0.05 versus L‐JWH133 group.

**Figure 5:**
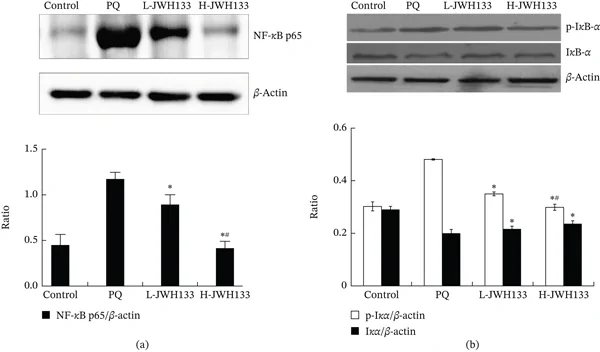
Effects of JWH133 on NF‐𝜅B signal transduction in lung tissues of PQ‐induced acute lung injury rats. JWH133 was intraperitoneally administered 1 h before intraperitoneal administration of PQ. Lung tissue was collected after PQ administration for 72 h to determine the expression of (a) NF‐𝜅B p65 and (b) I𝜅B‐α and p‐I𝜅B‐α by Western blotting. L‐JWH133: JWH133 at the low dose of 5 mg/kg and H‐JWH133: JWH133 at the high dose of 20 mg/kg. The values presented are the mean ± SD. ^∗^ *p* < 0.05 versus PQ group; ^#^ *p* < 0.05 versus L‐JWH133 group.

Please also find the full Western blots used to generate the new Figures at https://data.mendeley.com/datasets/46788gkvnw/1

We apologize for these errors.

